# Anther Transcriptome Analysis of Two Heat Tolerance-Differentiated Indica Rice Restorer Lines Reveals the Importance of Non-Structural Carbohydrates and ATP in the Regulation of Heat Tolerance

**DOI:** 10.3390/ijms26073161

**Published:** 2025-03-29

**Authors:** Jieqiang Zhou, Yingfeng Wang, Jiangfeng Li, Zijian Song, Yunhua Xiao, Huabing Deng, Xiong Liu, Qiuhong Chen, Wenbang Tang, Guilian Zhang

**Affiliations:** 1 College of Agronomy, Hunan Agricultural University, Changsha 410128, China; zhoujieqiang@stu.hunau.edu.cn (J.Z.); wangyf@stu.hunau.edu.cn (Y.W.); 15286442942@stu.hunau.edu.cn (J.L.); 2939362722@stu.hunau.edu.cn (Z.S.); yhxiao@hunau.edu.cn (Y.X.); denghuabing@hunau.edu.cn (H.D.); xiongliu@hunau.edu.cn (X.L.); cqh924@hunau.edu.cn (Q.C.); 2 State Key Laboratory of Hybrid Rice, Changsha 410128, China; 3 Hunan Hybrid Rice Research Center, Hunan Academy of Agricultural Sciences, Changsha 410128, China

**Keywords:** rice (*Oryza sativa* L.), heat stress, transcriptome, non-structural carbohydrate

## Abstract

Screening and breeding more resistant heat stress restorer lines represent an effective approach to addressing the decline in hybrid rice seed production caused by heat stress (HS). However, the molecular mechanisms affecting the differences in the heat resistance of anthers under HS remain unclear. This study compared the gene expression patterns of two hybrid rice restorer lines with differing heat resistances under HS and discusses the mechanisms of the heat response in rice. Under heat stress, 247 DEGs were co-expressed across varieties and were involved in biological processes such as protein processing and carbon metabolism, with heat shock proteins being the most ubiquitous. Interestingly, a substantial enrichment of genes related to non-structural carbohydrates and ATP was observed among the unique DEGs in R996 and R4628. Simultaneously, the contents of non-structural carbohydrates and ATP levels in the young spikes of R996 were significantly higher than those in R4628. This suggests that starch, soluble sugars and ATP play significant roles in heat tolerance during the flowering stage of rice. Overall, this study provides novel insights into the molecular mechanisms underlying heat stress resistance in indica rice restorer lines and informs future strategies for the genetic improvement of heat tolerance in these varieties.

## 1. Introduction

Environmental temperature is critical for crop growth and development. However, as global warming increases, the threat to crop yields from extreme heat is growing daily [1]. Rice (*Oryza sativa* L.), one of the world’s important food crops, is grown mainly in tropical and temperate regions and is being seriously threatened by global warming. It has been reported that for every 1 °C rise in global temperature, rice yields will be reduced by about 3.2% on average [1]. Therefore, it has become more important to gain a deeper understanding of the molecular mechanisms by which rice resists heat stress (HS).

HS always negatively affects plant growth and development and can lead to catastrophic crop yield reductions. HS affects all growth stages of rice, with the heading and flowering periods being the most temperature-sensitive. In agricultural production, reduced seed setting rate and poor filling due to HS at the flowering and heading stage are of greater concern than the apparent wilting that may occur during nutrient growth. During the flowering period, temperatures of higher than 35 °C for more than one hour may lead to rice sterility [2]. Under HS conditions, normal pollen development, abnormal anther dehiscence, restriction of spikelet opening and the degree of glume opening all prevent pollination and fertilization [3]. A heat treatment at 38 °C reduced the amount of pollen on the stigmas in *indica* variety N22 and the *japonica* variety Moroberekan by 55% and 86%, respectively [4].

Currently, a large number of HS-related genes and QTLs have been identified in rice. *Thermo-tolerance 1* (*TT1*) encodes the α2 subunit of the 26S proteasome and regulates HS tolerance in rice seedlings by degrading toxic denatured proteins under HS and maintaining the HS response process [5]. Two genes in the *TT3* locus antagonistically regulate the regulation of HS tolerance, with *TT3.1* antagonistically having a positive regulatory role and *TT3.2* antagonistically having a negative regulatory role [6]. *HTS1* encodes a β-ketolipoyl carrier protein reductase, and *HTS1* mutants accumulate more H_2_O_2_ and higher Ca^2+^ efflux upon heat treatment, thereby damaging cell membranes and chloroplasts [7]. In addition, a number of studies have shown that genes such as *OsNCED1*, *HTH5*, *OsANN1*, *SNAC3* and *OsRab7* can regulate HS tolerance in rice by maintaining ROS homeostasis [8,9,10,11,12].

Many studies have also found that the content of some endogenous metabolites or the application of exogenous substances can affect HS tolerance in rice. Brassinolide content in young panicles was reduced after HS treatment, and spraying 24-epibrassinolide was effective in mitigating the effect of spikelet degradation by HS [13]. The exogenous application of abscisic acid prior to HS reduces HS-induced pollen sterility by maintaining the carbon and energy balance [14]. Ethylene and hydrogen sulfide mediate HS acclimation in rice by regulating sulfur assimilation and photosynthesis [15].

The breeding of hybrid rice has made a significant contribution to world food security. As of 2019, China has bred more than 7000 hybrid rice varieties, with a total planted area of about 6 × 10^8^ hm^2^ and a total grain output increase of nearly 9 × 10^8^ t [14,16]. Therefore, it is important to breed hybrid rice with a high stress tolerance. However, the mechanism of the transcriptional regulation of genes in the anthers of hybrid rice restorer lines under HS treatment is currently unknown. Here, we have analyzed the transcriptome dynamics of anthers from two hybrid rice restorer lines, R996 and R4628, differing in HS tolerance, by RNA-seq. We sought to elucidate the key genes and pathways involved in the responses to HS in restorer line anthers, as well as to inform the understanding of the complex molecular response that occurs when anthers are stressed by HS.

## 2. Results

### 2.1. R996 Is More Resistant to HS than R4628

**Figure 1:**
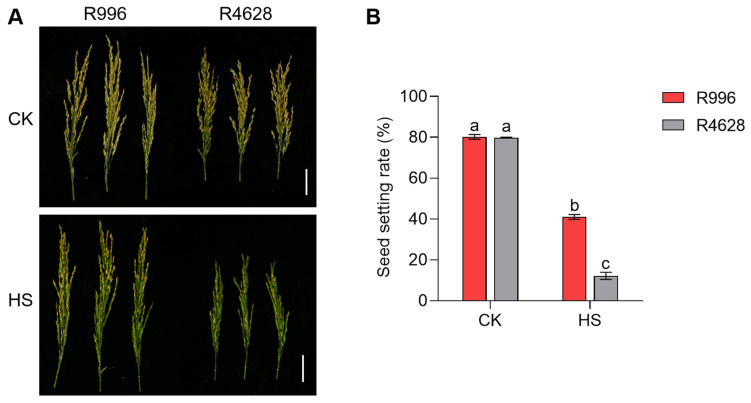
HS decreases plant seed setting rate. (**A**,**B**) Seed setting rates of R996 and R4628 under CK and HS. Three biological replicates were used for each plant. Data are means ± s.d. (*n* = 3). Different letters denote significant differences (*p* < 0.05) from a Duncan multiple-range test. Bars = 5 cm.

In order to investigate the effect of HS on the seed setting rate at the heading and flowering stages of hybrid rice restorer lines, we selected two indica rice restorer lines, R996 and R4628, to observe their fertility after HS treatment. R996 and R4628 showed different responses to HS (Figure 1). Under the CK conditions, there was no difference in the seed setting rate between the two rice plants. However, under HS, the seed setting rate of R996 was significantly higher than that of R4628 (Figure 1). This suggests that R996 possesses stronger HS tolerance compared to R4628 at the heading and flowering stages.

### 2.2. Decrease in Stigma Pollen Number and Loss of Pollen Viability Are Responsible for Reduced Seed Setting Rate Under HS

**Figure 2:**
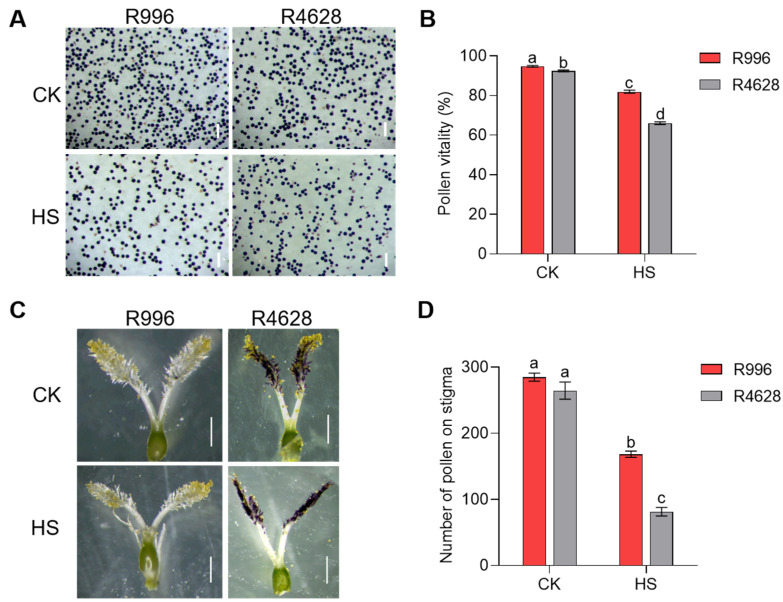
Statuses of R996 and R4628 pollen under HS. (**A**,**B**) Pollen vitality of R996 and R4628 under CK and HS. Bars = 200 μm. (**C**,**D**) Amount of pollen on stigmas of R996 and R4628 under CK and HS. Three biological replicates were used for each plant. Data are means ± s.d. (*n* = 3). Different letters denote significant differences (*p* < 0.05) from a Duncan multiple-range test. Bars = 1 mm.

When rice plants are affected by HS during the booting stage, this often leads to spikelet fertility decline due to decreasing pollen viability and inhibited anther dehiscence [17,18,19]. To clarify the reason for the difference in heat tolerance between R996 and R4628, the pollen viability and anther dehiscence rates of the plants were observed under CK and HS. Pollen activity is influenced by HS, and both R996 and R4628 showed significant decreases in pollen activity under HS, but the pollen activity of R996 was significantly higher than that of R4628 under the HS treatment (Figure 2A,B). In addition, we observed the anther dehiscence rates of R996 and R4628 and found that the anther dehiscence rate of R4628 was significantly lower than that of R996 under the HS treatment. In contrast, the difference in their anther dehiscence rates was not significant under the CK treatment (Figure S1).

We also found that the stigma pollen numbers of both varieties significantly decreased under the HS treatment relative to the CK treatment. Among them, R996 had significantly higher stigma pollen numbers than R4628 under the HS treatment, while both did not differ in stigma pollen numbers under the CK treatment (Figure 2C,D). These results indicate that HS had significantly less effect on the pollen and anthers of R996 than R4628.

### 2.3. RNA-Seq Results of Transcriptome Samples

To further analyze the possible molecular mechanisms underlying the differences in tolerance of HS between R996 and R4628 at the heading and flowering stages, we sequenced the transcriptomes of some anthers of the two restorer lines on the third day of the HS treatment. RNA sequencing (RNA-seq) data were generated from the rice anther samples under the different treatments. The transcriptome of each cultivar was analyzed using two treatments with three biological replicates for each treatment, and Illumina RNA-seq analysis of 12 samples yielded 82.02 G bp of data and 546,749,116 read pairs (clean reads) (Table S2). The total map ratio ranged from 89.82% to 95.98%, and the unique map ratio ranged from 84.19% to 86.65%; few reads could not be mapped to the reference genomes (13.35–15.81%).

To verify the reliability of the RNA-seq results, we randomly selected six genes for qRT-PCR. As shown in Figure S2, the trend of the RNA-seq results was consistent with that of the qRT-PCR results, indicating that the RNA-seq data were reliable and could be analyzed in the next step.

### 2.4. Differentially Expressed Gene Analysis

**Figure 3:**
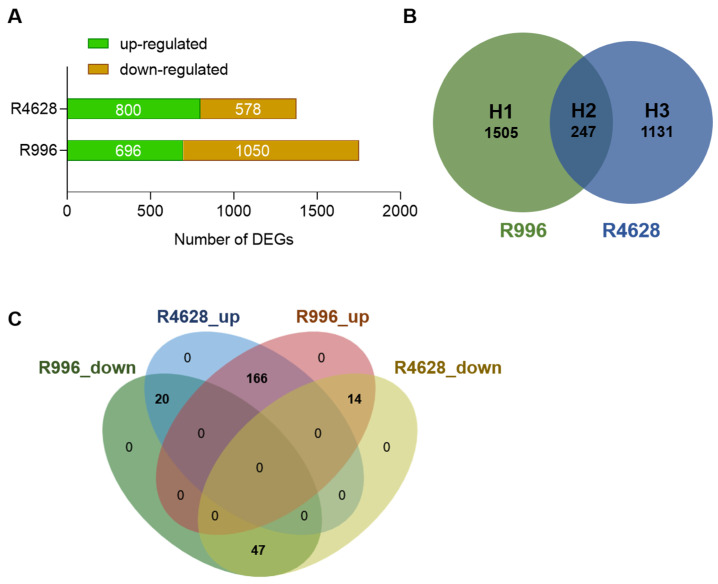
Statistical analysis of differentially expressed genes (DEGs) in R996 and R4628. (**A**) The numbers of DEGs in R996 and R4628, │log_2_FC│ ≥ 1, *q*-value ≤ 0.05. (**B**) Venn diagram comparison of total DEGs in R996 and R4628. (**C**) Venn diagram comparison of H2 DEGs in R996 and R4628.

To identify the DEGs in R996 and R4628 after the HS treatment, we selected │log_2_FC│ ≥ 1 and a *q*-value of ≤ 0.05 to screen for DEGs. The results, as shown in Figure 3A, showed that R996 had significantly more differential genes than R4628—1746 and 1378, respectively. Interestingly, there were significantly more downregulated DEGs in R996 than in R4628, while the opposite was true for upregulated DEGs. We then compared the expressed DEGs in the two varieties, which were categorized into three groups. There were 247 common HS-responsive DEGs (H2), 1505 DEGs specific to R996 (H1) and 1131 DEGs specific to R4628 (H3) (Figure 3B). In the H2 group, 14 DEGs were upregulated in R4628 but downregulated in R996, while 20 DEGs exhibited the opposite expression pattern (Figure 3C). Notably, four previously reported genes closely associated with pollen development—*Os03g0136400* [20], *Os05g0552300* [21], *Os08g0310100* [22] and *Os10g0189100* [23]—were identified among these DEGs, showing upregulation in R996 but downregulation in R4628.

### 2.5. Identification of TFs in DEGs

**Figure 4:**
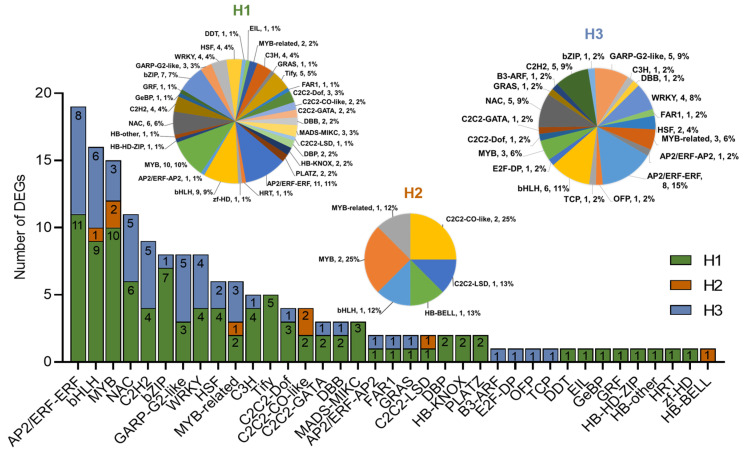
Transcription factor (TF) analysis of differentially expressed genes (DEGs) identified in H1, H2 and H3. Pie chart illustrating the distribution of TF families, represented by their respective numbers and percentages, among all DEGs. Bar plot displaying the number of genes associated with different TF families in H1, H2 and H3.

To better analyze the possible key genes for the difference in heat tolerance between R996 and R4628, we utilized the iTAK database to analyze the transcription factors (TFs) in the DEGs of H1, H2 and H3. Out of the 2408 TF genes belonging to 56 different TF families, a total of 160 differentially expressed TFs from 37 TFs families were identified, including AP2/ERF-ERF, bHLH, MYB, NAC, C2H2, bZIP, etc. Among these TFs, 99 were present in H1, 8 in H2 and 53 in H3 (Figure 4). There were 13 TF families identified only in H1, 1 TF family identified only in H2 and 4 TF families identified only in H3.

### 2.6. GO and KEGG Enrichment Analysis and PPI Network Construction of H2 DEGs

**Figure 5:**
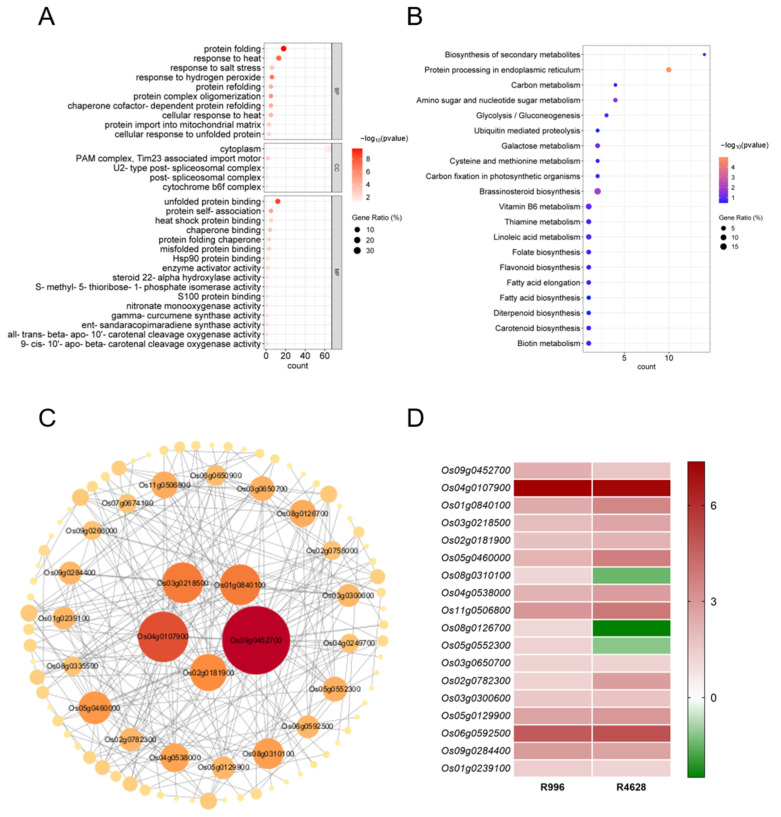
GO, KEGG and PPI analysis of DEGs in H2. (**A**) Gene Ontology (GO) annotation of DEGs in H2. The *Y*-axis represents the GO terms, while the *X*-axis represents the gene counts of the DEGs annotated to each GO term. (**B**) Kyoto Encyclopedia of Genes and Genomes (KEGG) pathway analysis of H2 DEGs. The *Y*-axis shows the names of the KEGG pathways. The *X*-axis shows the counts. The size and color of each point represent the Gene Ration and the −log_10_ (*p*-value), respectively. (**C**) Protein–protein interaction (PPI) networks of H2 DEGs were constructed by the STRING database. Each rectangle in the graph represents a gene, and the line indicates the relationship between the genes. All DEGs in the interaction network are calculated according to the degree, and the darker the color, the larger the unDir degree indicated. (**D**) The heat map shows the log_2_FC values of the top 18 DEGs in the H2 PPI network. These numbers indicate the log_2_FC values of the DEGs at the corresponding time points.

Next, we categorized GO annotations for the DEGs in the H2 group. In total, 157 GO terms were identified, including various biological processes (BPs), cellular components (CCs) and molecular functions (MFs). In particular, 93 GO terms were related to the BPs, 5 to the CCs and 59 to the MFs. As shown in Figure 5A, the GO terms for which the H2 DEGs were enriched included cytoplasm (GO:0005737, CC), protein folding (GO:0006457, BP), the response to organic substances (GO:0010033, BP), the response to oxygen-containing compounds (GO:1901700, BP), the response to heat (GO:0009408, BP) and unfolded protein binding (GO:0051082, MF).

KEGG pathway analysis was performed for the DEGs in group H2. The analysis showed that 247 DEGs were involved in 43 pathways. The DEGs of H2 were involved in protein processing in the biosynthesis of secondary metabolites; protein processing in the endoplasmic reticulum; carbon, amino sugar and nucleotide sugar metabolism; and brassinosteroid biosynthesis (Figure 5B).

To facilitate further identification of the genes located in the core positions, we constructed protein–protein interaction (PPI) networks of the H2 DEGs. The 93 DEGs with protein interactions were demonstrated in the PPI network of H2 (Figure 5C), while the expression patterns of the top 18 genes were illustrated in the heat map (Figure 5D). As illustrated in Figure 5C, heat shock proteins (HSPs) constituted the most prevalent class within the PPI network, such as Os04g0107900 (OsHSP1), Os01g0840100 (cHsp70-1), Os02g0758000 (OsHSP24.1), Os03g0245800 (OsHSP26.7), Os03g0266300 (OsHsp17.9A), etc. In addition, some non-HSPs may also play important role in HS tolerance at the heading and flowering stages, such as Os09g0452700 (UbL401), Os02g0181900 (OsClpB-m), Os08g0310100 (OsMRPL15), Os11g0506800 (OsBAG6), etc.

### 2.7. GO and KEGG Analysis of DEGs in H1 and H3

**Figure 6:**
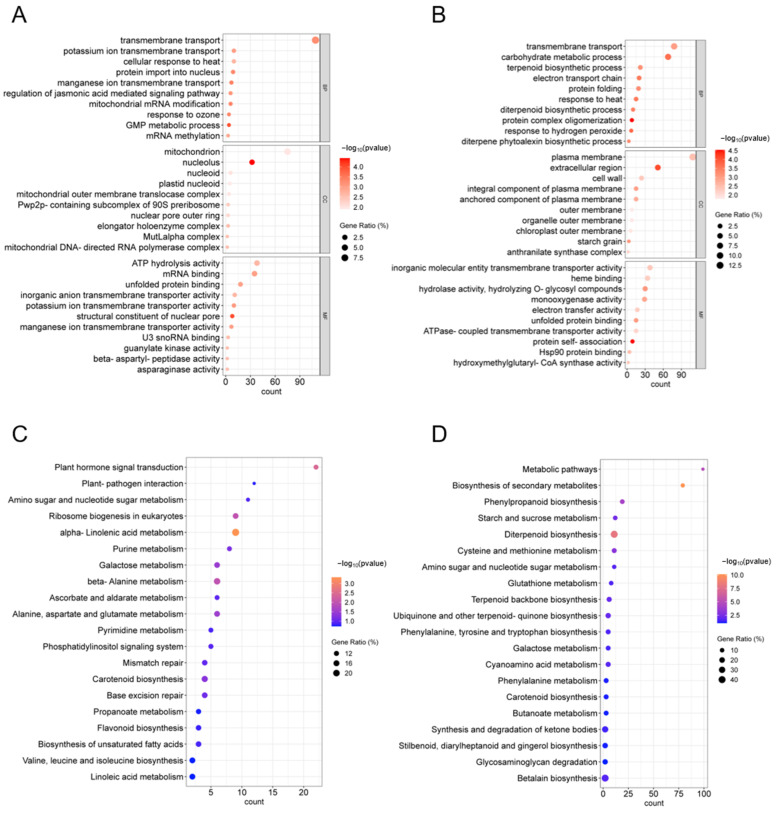
GO and KEGG analysis of H1 and H3 DEGs. GO analysis of H1 (**A**) and H3 (**B**). The *Y*-axis represents the GO terms, while the *X*-axis represents the gene counts of the DEGs annotated to each GO term. KEGG pathway analysis of H1 (**C**) and H3 (**D**) DEGs. The *Y*-axis shows the names of the KEGG pathways. The *X*-axis shows the counts. The size and color of each point represent the Gene Ration and the −log_10_ (*p*-value), respectively.

We present the top 10 GO terms for each group for H1 and H3 (Figure 6A,B). For the selected 1505 DEGs that were in H1, 1175 had GO annotations and were involved in 224 terms. The H1 DEGs involve transmembrane transport, the cellular response to heat, ATP hydrolysis activity and others (Figure 6A). For the 1131 selected DEGs that were H3, 849 had GO annotations and were involved in 231 terms. The H3 DEGs involve transmembrane transport, the carbohydrate metabolic process, carbohydrate transmembrane transporter activity, the response to heat, ATPase-coupled transmembrane transporter activity and others (Figure 6B). Notably, we found that both H1 and H3 enriched the most genes in transmembrane transport, while both H1 and H3 were enriched for the response to heat and ATP-related pathways.

To further investigate the DEGs involved in the various pathways in H1 and H3, KEGG enrichment analysis was performed for these two categories of DEGs. The DEGs of H1 and H3 were enriched into 105 and 86 KEGG pathways, respectively, and we show the top 20 KEGG pathways in Figure 6C,D. The DEGs of H1 are involved in plant hormone signal transduction, plant–pathogen interaction and amino sugar and nucleotide sugar metabolism, among others (Figure 6C). The DEGs of H3 are involved in metabolic pathways, the biosynthesis of secondary metabolites and phenylpropanoid biosynthesis, among which the metabolic pathways and secondary metabolite biosynthesis had the highest numbers of DEGs—99 and 79, respectively (Figure 6D). The KEGG pathways enriched for the DEGs in H1 and H3 were each not identical. Of interest is that many of these pathways are related to carbohydrate metabolism, such as amino sugar and nucleotide sugar metabolism, galactose metabolism, ascorbate and aldarate metabolism, starch and sucrose metabolism and others.

### 2.8. Non-Structural Carbohydrates and ATP Content

**Figure 7:**
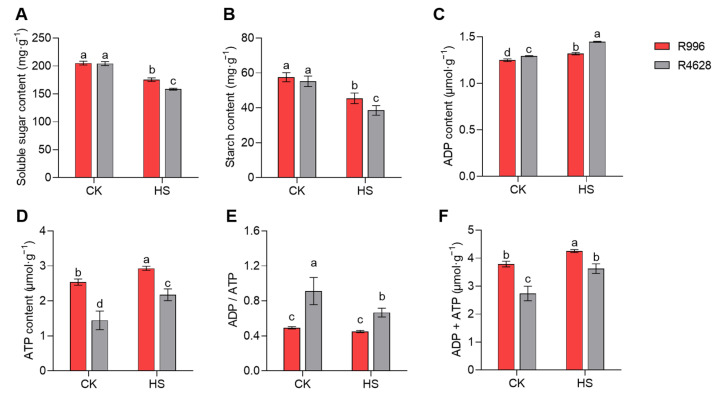
Effect of HS on carbohydrate and the ATP and ADP content in young panicles. Soluble sugar (**A**), starch (**B**), ADP (**C**) and ATP (**D**), content, as well as ADP/ATP (**E**) and ADP + ATP (**F**), in the young panicles of R996 and R4628 under CK and HS treatments. Three biological replicates were used for each plant. Data are means ± s.d. (*n* = 3). Different letters denote significant differences (*p* < 0.05) from a Duncan multiple-range test.

The GO and KEGG analyses of H1 and H3 showed that a large number of DEGs were enriched in carbohydrate metabolism-related pathways and ATP-related pathways in R996 and R4628, which strongly suggests that the carbohydrate and ATP contents of anthers may be responsible for the differences in the two restorer lines in terms of their tolerance to HS. Therefore, we first examined the soluble sugar and starch contents in R996 and R4628, which are two non-structural carbohydrates (NSCs), under different treatment conditions. The results showed that R996 and R4628 were not significantly different from each other in terms of starch and soluble sugar content under CK (Figure 7A,B). After HS treatment, both the soluble sugar and starch contents were significantly reduced in both varieties. Specifically, R4628 exhibited significantly lower contents than R996 in both soluble sugar and starch.

In addition, we also measured the ATP and ADP contents in the young panicles. Compared with the CK, the ATP and ADP contents in the young panicles of both varieties increased under the HS treatment, but the ATP content was significantly higher in R996 than in R4628, while the opposite was true for ADP (Figure 7C,D). Interestingly, R996 showed no significant change in ADP/ATP under the HS treatment, whereas R4628 showed a significant decrease (Figure 7E). We also noticed that the total amount of ADP + ATP was always significantly higher for R996 than for R4628 (Figure 7F).

## 3. Discussion

The flowering and filling stages are the most sensitive periods for young rice panicle growth, and either too-high or too-low ambient temperatures can lead to young panicle sterility and reduced seed setting rates [24]. Hybrid rice restorer lines are required to provide a large amount of pollen for hybrid rice seed production; therefore, the young panicle fertility of the restorer lines at extreme ambient temperatures is important. In this study, the seed setting rate of the restorer line R996 was significantly higher than that of R4628 under HS treatment, and we also found that the pollen viability, anther dehiscence rates and stigma pollen number of R996 also remained at high levels under HS treatment. These results suggest that R996 is more tolerant to HS and that anthers and pollen may be key factors.

The fragility of the protein structure leads to its susceptibility to damage by HS, resulting in misfolding or aggregation. In heat shock responses (HSRs), heat shock proteins (HSPs) are induced by heat shock factors (HSFs) and signaling molecules, which are responsible for protein refolding, assembly or degradation, thereby eliminating misfolded proteins [25]. In this study, we enriched a number of pathways related to protein binding and folding in R996 and R4628’s common DEGs. Also, a large number of genes encoding HSP were identified in the PPI network. *OsHSP1* is upregulated by heat stress, and heterologous overexpression in *Arabidopsis* enhances *Arabidopsis* HS resistance [26]. cHsp70-1 interacts with HsfA6a to synergistically regulate the transcription of *HSP101* [27]. Transgenic plants overexpressing *OsHsp17.9A* in tobacco had enhanced heat tolerance, while rice seeds silencing *OsHsp17.9A* using RNAi had reduced heat tolerance [28]. These results suggest that the HSP genes may play an important role in regulating the HS response in both varieties. The induction of the gene expression of HSPs and HSFs may be a common adaptive feature of rice cultivars in response to HS rather than specific to a particular cultivar, and these genes may be indispensable for HS resistance in rice. Furthermore, HSPs are likely to function as central components within the protein interaction networks activated under heat stress.

In this study, we found significant enrichment of carbohydrate-related pathways in both the H1 and H3 enrichment pathways. Meanwhile, our data also showed that the soluble sugar and starch contents of R4628 were lower than those of R996 under HS treatment. Consistent with our findings, Aqib et al. showed that heat-resistant cultivars accumulated more non-structural carbohydrates in panicles than heat-sensitive cultivars under HS treatment [29]. Carbohydrates have been shown to have a function in plant development and in the responses to different stresses. Starch is the primary carbohydrate in storage form, and when plant photosynthesis is limited, it releases sugars and other derivatives to provide energy and carbon and to support plant growth and resistance to stress during periods of stress [30]. On the other hand, soluble sugars can act as nutrients, osmoprotectants and metabolic signaling molecules in abiotic stress responses, among others [31]. Rice HSP60-3B interacts with FLO6 in plastids to mediate starch accumulation and viability in pollen and attenuates ROS levels in anthers, thereby ensuring normal development of rice male gametophytes under HS [32]. HS during microspore development affects floret fertility and pollen viability by influencing sugar translocation in different compartments of rice anthers and sugar utilization regulated by various genes and key enzymes for sugar conversion and carbohydrate metabolism [33]. In conclusion, the differences in the soluble sugar and starch contents in R996 and R4628 may be one of the possible reasons for the differences in HS tolerance.

There exists a close relationship between non-structural carbohydrates and ATP [30]. In our study, the GO and KEGG enrichment results indicated that many DEGs in H1 and H3 were associated with ATP (Figure 6), prompting us to focus on the relationship between ATP levels and heat stress resistance in plants. Under heat-stress conditions, plants enhance their heat tolerance by accumulating HSPs [34]. However, the accumulation of HSPs is an energy-intensive process. Under heat-stress conditions, the ATP synthesis rate in plants is adversely affected, leading to energy shortages that impede HSP accumulation and consequently compromise the plants’ thermotolerance [27,35,36]. Relevant studies have indicated that stress-resistant varieties possess higher ATP content compared with varieties that are more susceptible to stress conditions [34,37]. Rezaul et al. utilized ABA treatment on rice spikelets to elevate ATP levels, resulting in a significant enhancement of HSP gene expression levels and subsequently improving the heat tolerance of rice [38]. Furthermore, Wen et al. significantly elevated the ATP levels and ATPase activity in rice seedlings through the overexpression of *miR408*, thereby enhancing their thermotolerance [39]. These studies indicate that increasing the ATP levels in plants contributes to enhancing their thermotolerance. Consistent with the aforementioned research findings, our experimental results demonstrate that the ATP levels of R996 were significantly higher than those of R4628 after the heat treatment (Figure 7D). This suggests that the difference in the thermotolerance between the two varieties is attributable to the difference in the ATP levels, thereby implying a significant role of ATP in rice resistance to heat stress.

## 4. Materials and Methods

### 4.1. Plant Materials and Growth Conditions

This study was conducted in Changsha, Hunan Province, China. Two indica rice restorer lines were selected: namely, R996 and R4628.

Rice seeds were sown in a field and, 28 days later, transplanted into pots (each with a diameter of 266 mm and a height of 190 mm, housing three plants). The rice plants were then grown under natural conditions until they reached the heading stage. Once the primary panicles had developed to the booting stage; tillers that were heading (but not yet flowering) were identified and labeled. These plants were then placed in a plant growth chamber for a week to undergo heat stress (HS) treatments, while a control group (CK) was set up for comparison. After one week, all rice plants were transferred back to the CK conditions and incubated until they reached maturity and were harvested.

The HS treatment conditions were set at 37 °C (8:00–17:00) and 30 °C (17:00–8:00); the CK conditions were set at 30 °C (8:00–17:00) and 25 °C (17:00–8:00). The relative humidity was controlled at 75% during the treatment period, and the light intensity of the plant canopy was 850 µmol·m^−2^·s^−1^ with a 12 h (7:00–19:00) light duration.

### 4.2. Pollen Viability Determination

On the third day of the HS treatment, five spikelets that were poised to open either on the same day or the following day were selected. The anthers from each spikelet were carefully removed using tweezers and placed on slides for staining. A 1% I_2_–KI solution was then added for staining purposes. The average dyeability of the pollen was observed under a microscope across three sections, which served as an indicator of pollen viability.

### 4.3. Number of Pollens on Stigma Determination

On the third day of the HS treatment, ten stigmas (from five distinct plants) that had opened on that same day were selected for examination. The number of pollen grains adhering to each stigma was determined through microscopic observation and counting.

### 4.4. Anther Dehiscence Rate Determination

On the third day of the HS treatment, forty florets (from five spikelets) that had already bloomed were collected, two anthers from each floret were carefully removed using tweezers and placed on slides, anther dehiscence was observed under a microscope and the anther dehiscence rate was calculated.

### 4.5. Seed Setting Rate Determination

After seven days of the HS treatment, the plants were transferred to the CK temperature to grow until maturity. The seed setting rate was calculated and the experiment was replicated three times, with each replication consisting of two pots.

### 4.6. RNA Extraction

On the third day of the HS treatment, the florets that were about to open were collected. The anthers were promptly removed with forceps and placed in liquid nitrogen for rapid freezing. The total RNA was extracted using TRIzol in accordance with the manufacturer’s instructions (Invitrogen, Carlsbad, CA, USA) [40].

### 4.7. RNA-Seq Data Processing

RNA-seq libraries were prepared from anthers of R996 and R4628 plants subjected to heat stress (HS) and control (CK) treatments, respectively. Each treatment included three replicates, resulting in a total of 12 libraries. These libraries were sequenced individually using the BGISEQ-500 sequencer. In all samples, a *q*-value of ≤ 0.05 and an absolute value of │log_2_ fold change│ (│log_2_FC│) ≥ 1 were used to identify differentially expressed genes.

### 4.8. Analysis of DEGs

To understand the functions of the differentially expressed genes (DEGs), we conducted Gene Ontology (GO) enrichment analysis and Kyoto Encyclopedia of Genes and Genomes (KEGG) enrichment analysis of the DEGs using the R package “PlantNGSTools” (https://github.com/biomarble/PlantNGSTools, accessed on 7 June 2024). Sequences of the transcription factors (TFs) were searched in the iTAK database (http://itak.feilab.net/cgi-bin/itak/index.cgi, accessed on 7 June 2024) and compared to differential gene sequences. We used SIRING (https://string-db.org/, accessed on 7 June 2024) to analyze protein–protein interaction (PPI) networks and Cytoscape v.3.10.1 to draw the regulatory network of target genes. A bubble chart was plotted by https://www.bioinformatics.com.cn (last accessed on 10 December 2024), an online platform for data analysis and visualization.

### 4.9. Validation of DEGs by qRT–PCR

The total RNA was extracted as described previously and reverse-transcribed using the HiScript II Q RT SuperMIX for qPCR (+gDNA wiper) kit (R223-01, Vazyme, Nanjing, China). The qRT-PCR experiments were analyzed using the Hieff^®^ qPCR SYBR Green Master Mix (Yeasen, Shanghai, China), and the 2^−ΔΔCt^ algorithm was used to calculate the gene expression levels. The specific primers utilized are outlined in Table S1.

### 4.10. Non-Structural Carbohydrate Measurements

Young panicles were taken on the third day after HS treatment for soluble sugar content and starch content determination. The young panicles were ground to powder in liquid nitrogen and incubated with 80% ethanol at 80 °C for 30 min to separate the soluble sugars and starch. Determination of starch content (BC0700) and soluble sugar content (BC0030) was carried out according to the lab manual from the Solarbio kit.

### 4.11. ATP and ADP Content Measurements

Young panicles were taken on the third day after HS treatment for ATP and ADP content determination. The ATP and ADP contents were determined using ATP and ADP assay kits according to the manufacturer’s instructions (Shanghai Enzyme-linked Biotechnology Co., Ltd., Shanghai, China). During this process, 0.1 g of frozen young panicles were homogenized with 1 mL of 0.1 M PH7.4 PBS in an ice bath and centrifuged at 3000× *g* for 20 min. The supernatant was collected for analysis at 450 nm.

## Data Availability

The original contributions presented in this study are included in this article and the Supplementary Materials; further inquiries can be directed to the corresponding author.

## Supplementary Materials

The supporting information can be downloaded at: https://www.mdpi.com/article/10.3390/ijms26073161/s1.
