# Supplementary material for: Anther Transcriptome Analysis of Two Heat Tolerance-Differentiated Indica Rice Restorer Lines Reveals the Importance of Non-Structural Carbohydrates and ATP in the Regulation of Heat Tolerance
Source: Int J Mol Sci. 2025 Mar 29;26(7):3161. doi: 10.3390/ijms26073161 (PMC11989966; doi:10.3390/ijms26073161)
Supplement: Supplementary file 1 [file ijms-26-03161-s001.zip › Supplementary Figure.pdf]

Supplementary figures

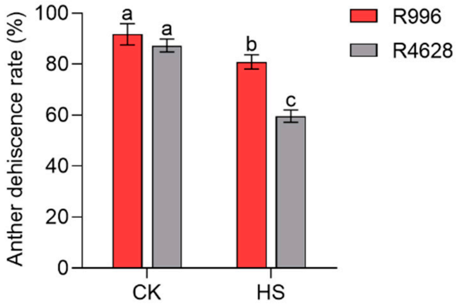

Figure S1. HS affects anther dehiscence. Data are means ± s.d. (n = 3). Different letters denote significant differences ($P < 0.05$) from a Duncan's multiple range test.

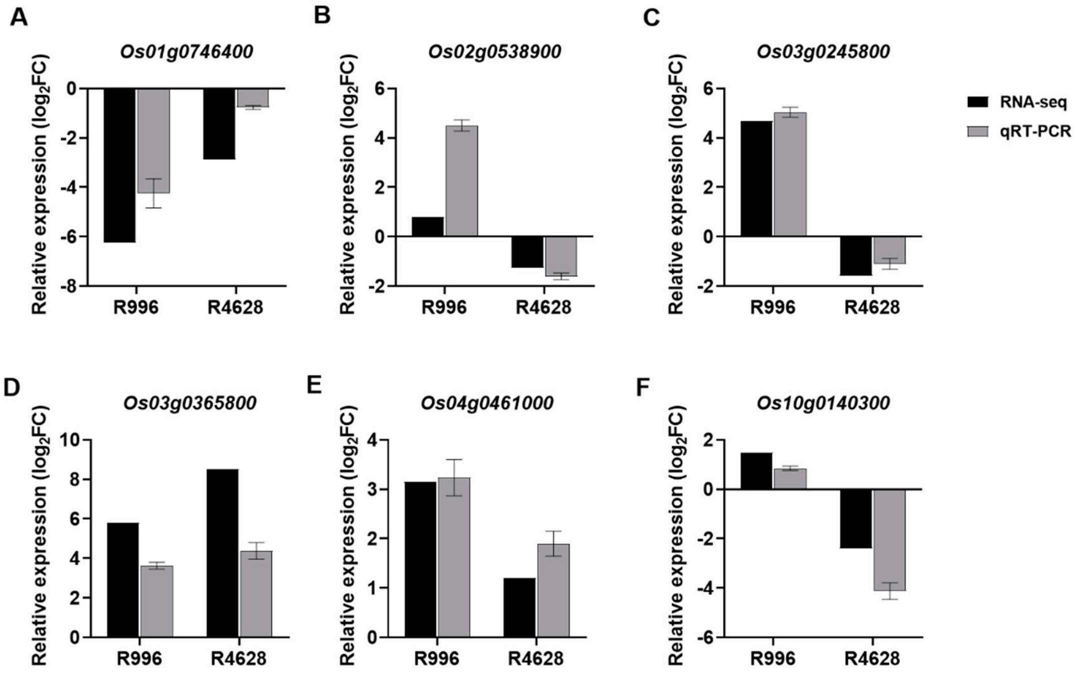

Figure S2. qRT-PCR verification of genes expression profiles in RNA-seq. Changes in gene expression levels (log2 FC values) of (**A**) *Os01g0746400*, (**B**) *Os02g0538900*, (**C**) *Os03g0245800*, (**D**) *Os03g0365800*, (**E**) *Os04g0461000*, and (**F**) *Os10g0140300* in qRT-PCR and RNA-seq. Data are means ± s.d. (n = 3).
